# Rapid Chairside Microbial Detection Predicts Endodontic Treatment Outcome

**DOI:** 10.3390/jcm9072086

**Published:** 2020-07-03

**Authors:** Alan Knight, Ian Blewitt, Nassr Al-Nuaimi, Tim Watson, Dylan Herzog, Frederic Festy, Shanon Patel, Federico Foschi, Garrit Koller, Francesco Mannocci

**Affiliations:** 1 Department of Endodontics, Faculty of Dentistry, Oral and Craniofacial Sciences, Floor 22 Tower Wing, Guy’s Dental Hospital, London SE1 9RT, UK; Alan.e.knight@kcl.ac.uk (A.K.); i.blewitt@nhs.net (I.B.); nassr.alnuaimi@gmail.com (N.A.-N.); timothy.f.watson@kcl.ac.uk (T.W.); dylan.b.herzog@kcl.ac.uk (D.H.); frederic.festy@kcl.ac.uk (F.F.); shanon.patel@kcl.ac.uk (S.P.); federico.foschi@kcl.ac.uk (F.F.); Garritt.koller@kcl.ac.uk (G.K.); 2 Department of Conservative Dentistry, College of Dentistry, University of Baghdad, Baghdad 10001, Iraq; 3 Specialist practice, London W1G 8SB, UK; 4 Department of Therapeutic Dentistry, I. M. Sechenov First Moscow State Medical University, 119146 Moscow, Russia

**Keywords:** endodontics, clinical outcomes, bacteria, biofilm(s), clinical studies/trials, computed tomography

## Abstract

Background. The aim of this longitudinal, one-year cohort study was to explore the hypothesis that fluorescence sampling of the root canal space prior to obturation could predict the outcome of root canal treatment (RCT). Methods. Sixty-five teeth underwent primary RCT and were followed up clinically and radiographically. The outcome was determined radiographically with periapical radiographs (PR) and cone beam computed tomography (CBCT) scans. Results. Success at 12 months was predictable based on the fluorescence score. When the fluorescence score (defined as the percentage of signal over total signal including background) was lower than 67, there was a 4.5 times (Odds ratio (OR) = 0.028; 95% confidence interval (CI): 0.003, 0.291, *p* = 0.001) greater chance of success (90% overall). When the readings were above this threshold, the success rate was 20%. Conclusion. A chairside sampling method is able to predict the outcome of RCT, through the use of paper point sampling and fluorescence staining. This has reduced the prevalence of persistent infections by guiding the optimum time for obturation. ClinicalTrials.gov trial NCT03660163.

## 1. Introduction

Root canal treatments (RCTs) are undertaken to eradicate bacterial infections from root canals (RCs) as persistent infections are associated with failure of the RCT [1,2]. An ability to detect bacteria still present in the root canal space will inform the need to continue disinfection through additional instrumentation and irrigation. This process would be completed with the aim of reducing the bacterial load, which in turn would increase the success rate of the RCT. Conventional techniques for bacterial detection and quantification such as colony-forming unit (CFU) counting [3] and DNA extraction, followed by polymerase chain reaction using universal bacterial primers [4] are time-consuming and not practicable for use in routine RCTs. Our group has developed and validated a methodology based on the use of a fluorescent vital dye for the chairside detection of vital cells present in the canal space, sampled with sterile endodontic paper points during RCTs [5]; this technique was found to be more sensitive than bacterial culture in detecting bacteria in vitro and in vivo [5].

Periapical radiography (PR) is the technique of choice for assessing the outcome of endodontic treatments [6,7]; however, periapical radiolucent lesions are usually only diagnosed when there has been perforation or erosion of the overlying cortical plate [8,9]. Cone Beam Computed Tomography (CBCT) was found to be more accurate than periapical radiography in detecting the presence of apical periodontitis in studies where fresh human cadavers were used as the reference standard [10,11]. At one-year recalls, using loose criteria approximately 25% of RCT in molars fail when assessed using CBCT [12] and require further interventions such as re-RCT, apicectomy or extraction: a large proportion of these failures are likely to be associated with the persistence of bacteria within the root canal space.

The aim of this study was to evaluate using CBCT the outcome of RCT in relation to the result of a pre-obturation paper point sample, establishing a cut-off fluorescence level able to predict the outcome of RCT.

## 2. Materials and Methods

### 2.1. Study Design and Patient Recruitment

Ethical approval was sought and gained by the NRES London Bridge and Dulwich Research Ethics (REC Ref. 10/H0804/56 and 08/H0804/79). The study was registered on ClinicalTrials.gov (NCT03660163). The study followed the recommendations of the STROBE statement for observational epidemiology studies [13]. In this study, 81 patients undergoing primary root canal treatments were recruited as a part of a larger clinical trial on root canal treatment outcome; verbal and written informed consent was taken from all patients. In total, 100 teeth were recruited consecutively between September 2015 and December 2016. Treatments were carried out by 10 postgraduate endodontic residents. Recruited patients were not involved with any previous clinical trial.

The teeth included in the study had been referred to a specialist centre for the treatment of irreversible pulpitis or pulp necrosis. Patients were excluded if they were pregnant, immunosuppressed, had non-restorable teeth or teeth with periodontal probing depth >3 mm. Prior to endodontic treatment, all teeth were subjected to vitality testing.

Methods of pulp assessment included pulp sensibility, palpation and percussion tests, along with the presence of pain, abscess, sinus tract and abnormal mobility. Sensibility tests undertaken included thermal (Endo-Frost; Roeko Coltène/Whaledent, Langenau, Germany) and electric pulp testing (Kerr Vitality Scanner 2006; SybronEndo, Orange, CA, USA).

### 2.2. Endodontic Treatment and Intraoperative Sampling

Root canal treatment was performed by postgraduate residents under the direct supervision specialist endodontic staff at King’s College London Dental Institute. All operators were trained and provided with a standardised step-by-step treatment protocol to adhere to. The CBCT scans and PR were available prior to and during RCT. All treatments took place over two visits. During the first visit, the tooth requiring treatment was anaesthetised, dental plaque and calculus removed and the tooth surface cleaned with pumice. Prior to application of the rubber dam, all caries and existing restorations were removed to assess the restorability of the tooth. If necessary, the tooth was built-up with glass ionomer cement (Fuji IX; CG America, Alsip, IL, USA) prior to rubber dam isolation. A dental operating microscope (3 step entrée; Global, St. Louis, MO, USA) and ultrasonic instruments were used to aid canal identification.

Pre-treatment, the mouth was disinfected using 0.2% chlorhexidine, followed by placement of a rubber dam which was also disinfected with 3% sodium hypochlorite (NaOCl, Parcan, Septodont, Lancaster, PA, USA) to avoid contamination of the samples; the tooth surface was also disinfected with NaOCl. Root canals were prepared with hand files to at least a size 20 prior to using ProTaper Next nickel-titanium rotary instruments (Dentsply Sirona, Ballaigues, Switzerland) to a minimum of apical size 25 (ProTaper X2) by the end of the second visit. Canals were continuously irrigated with 3% sodium hypochlorite throughout the procedure, which took 60 min on average. After shaping, the canals were dried and calcium hydroxide dressing (Hypocal^®^, Ellman International, Oceanside, NY, USA) was placed for a minimum of 10 days. A temporary restoration (IRM; Dentsply Sirona) was placed to seal the tooth. In the second visit, the tooth was anesthetised and isolated with a rubber dam, and access was regained, following the aforementioned disinfection protocol, used in the first visit. The canals were reirrigated, again with sodium hypochlorite, and a further 17% ethylenediaminetetraacetic acid (EDTA; Pulpdent, Watertown, MA, USA) irrigation to remove the smear layer. Passive ultrasonic irrigation was then performed in each canal for 1 min. A penultimate rinse with EDTA was undertaken followed by a final rinse with sodium hypochlorite. 

All sampling procedures were undertaken by one investigator. Endodontic paper points were used to sample the RC space pre-obturation. For every sampled tooth the rubber dam prior to testing was swabbed with sterile saline and sterile paper points were scrubbed on the rubber dam and tested as controls.

Before sampling with paper points, the RC space was thoroughly rinsed using sterile saline solution to remove any remaining irrigant solutions used during treatment (NaOCl and EDTA). Each canal was then sampled with two paper points to remove excess moisture and collect the maximum amount of residual bioburden. Samples were then assessed using a fluorescent spectral analysis as described by Herzog et al. [5]. Calcein AM is a vital fluorescent stain, which only becomes fluorescent in the presence of vital cells. In brief, immediately after sampling, the paper points were stained in 1.5 mL polypropylene tubes containing a phosphate buffered solution (PBS) of Calcein AM (10 μm) and incubated for 5 min at room temperature in a dark environment, before rinsing with PBS. For every sampled tooth, stained sterile paper points from the same package were also tested as a quality control to both provide a background spectrum and aseptic control. Finally, the paper points were pressed between two sterile microscope slides, removed and transferred into glass bottom well plates. Five fluorescent spectra were recorded using a 480 nm light spectrometer; readings were taken at 500 μm intervals, starting from the apex, before being analysed using spectral unmixing based on linear regression [5]. This method allowed the normalised quantification of the vital signal in Arbitrary Units (AU) from the Calcein to be recorded using the formula:

Fluorescence Reading (%) = Calcein Signal/(Calcein Signal + Background Autofluorescence) × 100

As the detection was strongest at the paper points tip, the first three measurements from the tip were averaged and the value gained from the proportional Calcein fluorescence was used as an indicator of the percentage score [5].

For multi-rooted teeth, the value of the highest reading was used for the prediction of the outcome. Canals were then filled with gutta-percha and AH Plus (Dentsply Sirona) using a continuous wave condensation technique. Teeth were restored with a permanent composite core (Herculite^®^ Ultra, Kerr Corporation, Orange, CA, USA). Patients were referred back to their dentist for indirect cuspal coverage restorations.

### 2.3. Clinical and Radiographic Assessments

All teeth were assessed clinically and radiographically at baseline (T0) and 12 months after completion of RCT (T12). All patients underwent a preoperative pain history and examination that included assessment of the tooth for tenderness to percussion, mobility and increased probing depths. Soft tissues were assessed; the presence of sinus tracts and tenderness to palpation was noted. Data were anonymised onto spreadsheets (Microsoft Excel 2011; Microsoft^®^, Redmond, WA, USA).

Periapical radiographs and CBCT scans were taken pre- and one-year postoperatively. Preoperative PRs using the paralleling technique were obtained with digital phosphor plate system (Digora^®^; Optime, Soredex, Tuusula, Finland) using a beam positioning film holder (Dentsply Rinn, Elgin, IL, USA). All radiographs were exposed using a dental X-ray machine (Heliodent, Sirona, Bensheim, Germany) operating at 65 kV, 7 mA and an exposure time of 0.16–0.25 s. A small volume CBCT scanner (3D Accuitomo 80; J. Morita, Kyoto, Japan) with a 4 cm × 4 cm field of view, 0.125 mm of voxel size, 90 kV, 4 mA and 17.5 s was used to obtain CBCT images. All CBCT data were reconstructed using the system’s proprietary software (i-Dixel^®^ 3DX; J. Morita, Kyoto, Japan) with 0.16-mm slice intervals and 1.2 mm slice thickness. Patients presenting with clinical symptoms before the recall date were classified as failures.

The CBCT images were selected by a blinded calibrated examiner. Slices were assessed and selected to demonstrate the largest extent of the periapical lesion. Each root was vertically aligned using Accuitomo software (One Volume Viewer^®^, J. Morita Kyoto, Japan). All identifying patient information was removed. The blinded radiographs at T0 and T12 and CBCT images were displayed together as PowerPoint (Microsoft^®^) presentations using two 13-inch laptop computers (MacBook Pro, Apple, Cupertino, CA, USA) with a resolution of 1680 × 1050 pixels to allow the examiners to determine the healing status of each root. The raw data were also available to allow the examiners to measure the largest extent of the periapical lesion. The radiographic interpretation was conducted in a quiet, dimly lit room. A consensus panel of two pre-calibrated experienced endodontists was asked to identify the presence, absence or change (increase/decrease) in the size of an existing periapical radiolucency associated with the apical portion of the roots. The examiners were not involved in the endodontic treatments. For calibration, the examiners were asked to assess the healing status in 20 examples of one-year follow-up of PRs and CBCT images of root filled teeth that did not belong to the present experimental material. Furthermore, each examiner was asked individually to assess the pairs of the PRs and CBCT images a second time after four weeks to determine the inter-examiner reliability.

The assessment of radiographic images was performed in two viewing sessions, at least one week apart. During each session, the consensus panel was asked to assess 50% of the PRs and 50% of CBCT scans. The consensus panel reliability was determined by jointly re-assessing 20 pairs of the preoperative and one-year postoperative radiographic images following a two-week interval.

In multi-rooted teeth, the periapical status of each root was established. This allowed a direct comparison of like-pairs of specific roots using radiographs and CBCT images as described in [12]. The outcome of the tooth was determined by the root with the worst periapical status.

A periapical lesion was defined when a periapical radiolucency extended beyond twice the width of the periodontal ligament space [14,15]. Based upon the radiographic changes between T0 and T12 images, each tooth was allocated to the most appropriate outcome category (Table 1).

In the present study, teeth with healing lesions (Outcome 4), healed lesions (Outcome 5) or an unchanged healthy periapex (Outcome 6) were considered to have a “favourable” outcome, whereas teeth that developed a new lesion (Outcome 1), teeth with an increase in size of existing lesion (Outcome 2), no change in the existing lesion size (Outcome 3) and teeth with symptoms and/or clinical signs of failure (i.e., tenderness to percussion and/or palpation, presence of swelling and sinus tract), irrespective of the radiographic outcome, were considered to have an “unfavourable” outcome.

Postoperative root filling length was assessed on CBCT and dichotomised as adequate (0–2 mm short of the radiographic apex) or inadequate short or long (>2 mm short of the radiographic apex or beyond the radiographic apex). The quality of the restorations placed on the recalled teeth was assessed clinically and radiographically and classified into acceptable and not acceptable.

### 2.4. Statistical Analysis

Data analysis was carried out using IBM SPSS software (version 23: IBM, New York, NY, USA). Fleiss’s kappa coefficient was used to assess the intra-consensus panel agreement and inter-examiner agreement for radiographic assessments of periapical health and obturation length. The outcome of root canal treatments was dichotomised into “favourable” versus “unfavourable”, whereas the apical extension of the filling was dichotomised into “adequate” versus “inadequate”.

To determine the association between fluorescence reading value and the outcome of RCT, fluorescence readings (AU) were analysed by means of receiver operating characteristic (ROC) curve and Youden’s index (sensitivity + specificity − 1) to identify the optimal cut-off point for fluorescence reading dichotomisation.

Chi-square (χ^2^) tests were used to determine the association between the outcome and other variables (fluorescence reading and apical extension of root canal filling). If the chi-square test showed that any of these variables were significant, then the joint association of these variables with the treatment outcome was tested using a logistic regression model. The significance level was set at 0.05. A McNemar’s test was used to determine if there were any significant differences in favourable and unfavourable outcomes of treatments when assessed by PR and CBCT. Fisher’s exact test was used to determine whether there was any significant association between the preoperative periapical status and the outcome of treatment.

## 3. Results

**Figure 1:**
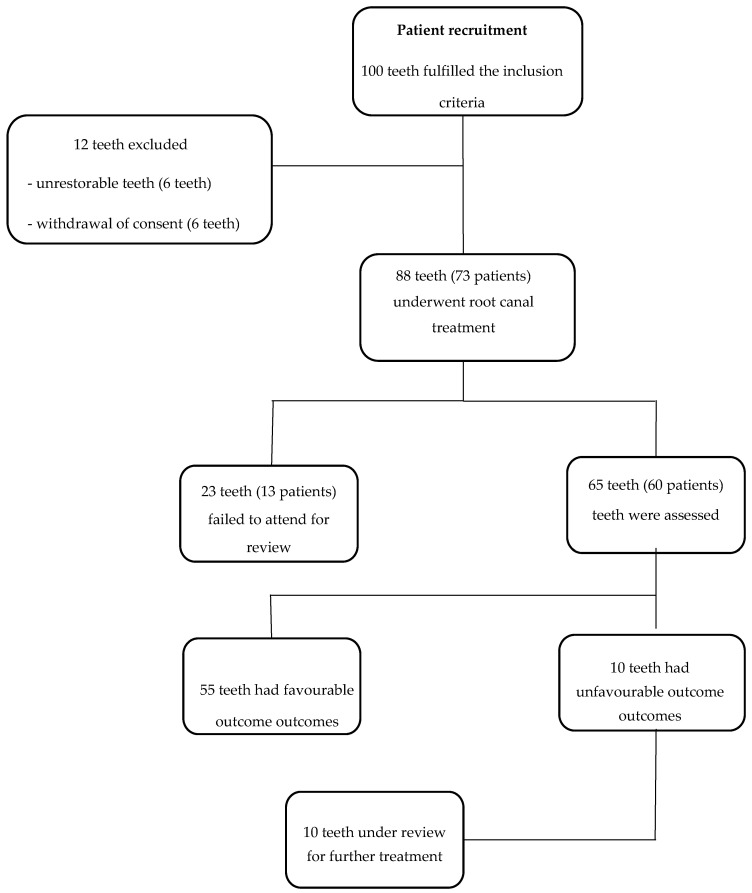
Flowchart of the trial showing the process of patient recruitment, exclusion and follow-up.

A flowchart of the trial showing the process of patient recruitment, exclusion and follow-up is presented in Figure 1. Patients’ age ranged between 17 and 78 years, with an average age of 45. Thirty patients were male (50%) and 30 were female (50%). There were 13 anterior teeth treated, 8 premolars and 44 molars. Of the 88 teeth which underwent root canal treatment, 65 were reviewed with a recall rate of 74%. At the one-year review, all patients were asymptomatic.

Of the 65 teeth reviewed at one year, 55 had been classified as “healing” and 10 as “non-healing” following CBCT imaging. Only eight of the 65 recalled teeth did not show a CBCT apical radiolucency at baseline.

No fluorescent reading was detected on the paper points used to detect contamination from the rubber dam.

Using “loose” success criteria and CBCT assessment, 55 cases were deemed to have a favourable outcome (including healing and healed teeth), an 84.6% success rate. Using “strict” (healed) criteria, 32 teeth healed, a 49% success rate.

**Figure 2:**
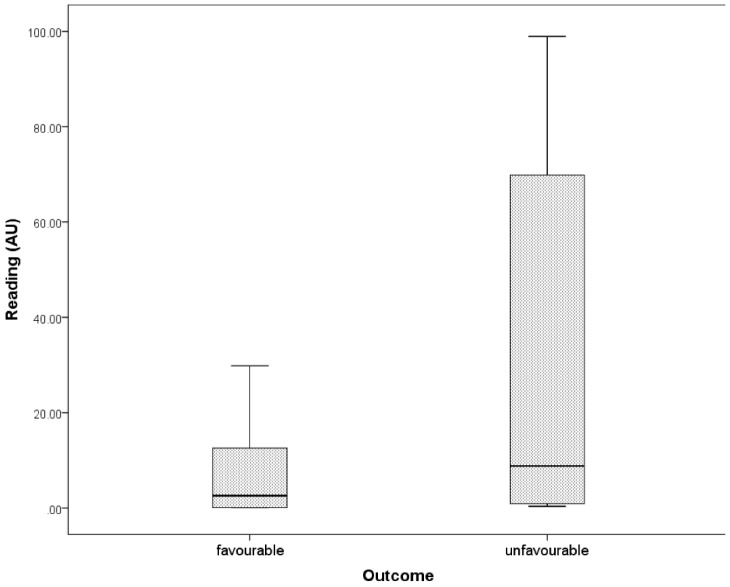
Levels of fluorescence reading (AU) reflects patients’ (*n* = 65) response to endodontic treatment according to “loose” success criteria. The probability of 12-month success was 4.5 times (Odds ratio (OR) = 0.028; 95% confidence interval (CI): 0.003, 0.291, *p* = 0.001) higher when the fluorescence reading was lower than 67 (%) with success rates of 90% and 20%, respectively, for teeth with readings below and above this threshold.

Based on Youden’s index associated with ROC curve, the optimal cut-off point of fluorescence reading was 67 (AU) and the fluorescence readings were dichotomised accordingly. The percentage of the difference was statistically significant (Fisher’s exact test *p* = 0.001). The probability of 12 months success was 4.5 times (Odds ratio (OR) = 0.028; 95% confidence interval (CI): 0.003, 0.291, *p* = 0.001) higher when the fluorescence reading was lower than 67% with success rates of 90% and 20%, respectively, for teeth with readings below and above this threshold (Figure 2). With readings <67, 90% (*n* = 54) of teeth had a favourable outcome, whereas a favourable outcome was only recorded in one case where the fluorescence reading value was >67.

The intra-consensus panel agreement and the inter-examiner agreement for assessing the treatment outcome was very high at 0.92 for both PR and CBCT.

At T12 recall assessment, PR revealed the healing outcome (4) and healed rate (5 and 6) were 20% (13) and 70.8% (46), respectively, with an overall favourable outcome of 90.8%. With CBCT, the healing outcome (4) and healed rates (5 and 6) were 41.5% (27) and 43.1% (28), respectively, with an overall favourable outcome of 84.6%.

There were 10 teeth recorded as having an unfavourable outcome when assessed with CBCT (15.2%) and six teeth by PR (9.2%).

With preoperative apical periodontitis, a favourable outcome with PR and CBCT was 87.7% (50) and 82.8% (48), respectively. However, if there was no preoperative apical periodontitis, a favourable outcome was reached in 100% with both PR and CBCT (Table 2).

With PR, a higher success rate was seen in molars (90.9%) compared to anterior teeth (84.6%). However, with CBCT, there was no difference in outcome between anterior teeth (84.6%) and molar teeth (84.1%). Of the 10 teeth that were deemed as an unfavourable outcome, seven were molars, one was a premolar and two were incisors.

When looking at the length of obturation, the biggest difference between inadequate (short and long) and adequate obturation was in the molar group of teeth, where 91.9% (34/37) of teeth had a favourable outcome with adequate obturation and only 42.9% (3/7) with inadequate obturation. Overall, 89.1% (49/55) had a favourable outcome if there was an adequate length vs. 60% (6/10) if the length of obturation was inadequate (Table 3).

Sixty out of 65 recalled teeth had been restored with full gold or metal-ceramic crowns, 64 out of the 65 recalled restorations were classified as acceptable both clinically and radiographically, all the failed teeth had acceptable restorations and only one of them had not been covered with a crown.

## 4. Discussion

This study demonstrates that the outcome of root canal treated teeth can be predicted through the use of fluorescence. The teeth that had a higher fluorescence score (>67) were more likely to fail when compared to teeth with a low score (<67).

Youden’s index is often used in conjunction with receiver operating characteristic (ROC) analysis. The index is defined for all points of a ROC curve, and the maximum value of the index may be used as a criterion for selecting the optimum cut-off point when a diagnostic test gives a numeric rather than a dichotomous result. The index is represented graphically as the height above the chance line, and it is also equivalent to the area under the curve subtended by a single operating point.

The proportion of high readings progressively increased going from the “healed” to the “healing” category, reaching its highest value (60%) in the “failed” group. Teeth with values >67 had a 4.5-fold greater chance of unfavourable treatment outcome at T12 than those that had a lower fluorescence reading (<67). These results were comparable with those achieved in microbiologic studies correlating the results of bacterial culture of paper-points samples taken at the end of the root canal treatment with the outcome of RCT assessed using PR with five-year recall [3]. Calcein AM was also found to be more sensitive than bacterial culture in detecting bacterial biofilms [5]. The combined use of CBCT and rapid microbial detection significantly increased the power of this study.

The sampling technique was validated in a previous study on patients [5], in which conventional culturing techniques and fluorescence detection were used in the same patients with comparable results. In a preliminary study by Herzog et al. (unpublished), the use of neutralising agent such as sodium thiosulfate did not affect the result of bacterial sampling and therefore its use was not included in the sampling protocol. After application of the Calcein-AM solution to the paper sampling points, a kinetic assay was carried out to determine the increase in fluorescence arising from ester hydrolysis of viable cells, converting the non-fluorescent substrate to a brightly fluorescent, intracellular product, which was detected using the Saferoot device. After equilibration for approximately 30 s, measurements were taken every 30 s and intensity plotted. Paper points showing no vital material did not significantly yield hydrolysis (and increasing fluorescence) over the 5-min study period, whilst a linear increase was observed for the contaminated samples and the relative increase recorded for each measurement.

In a small proportion of cases, high fluorescence reading values did not predict the failure of RCT. This could be because microorganisms were entombed within a well obturated RCS. All the failed teeth showed adequate coronal restorations at recall; it is, therefore, unlikely that the unpredicted failures are associated with coronal leakage. Ultimately, the small number of root canal treatment failures within the trial does not permit to evaluate the effect of the root canal obturation technique on the success and failure rate.

The use of CBCT to assess the outcome of RCTs allowed the detection of a much higher number of endodontic failures compared to PR [12,16,17]. The main objective of the present investigation was to establish an association between the fluorescence reading before obturation and the outcome of the root canal treatment; in this respect, the baseline level of infection of the teeth was not particularly important, however only eight teeth without initial radiolucency were included in the trial, hence the vast majority of the teeth treated were necrotic and infected.

The results were evaluated using CBCT and periapical radiographs at one year as in previous studies [12,16,17]. The fluorescence readings were able to predict the “favourable” outcome, which includes both complete and incomplete healing. It has been demonstrated in previous periapical radiograph-based studies that most “healing” cases progress to complete healing in successive recalls [18].

The use of a vital fluorescent stain allows the clinician to predict the outcome of RCTs with increased confidence, and, where residual bacteria are found at the end of the RC disinfection, to take additional measures to reduce the bacterial load below the safe threshold. Such additional measures might include further enlargement of the apical preparation, use of different irrigants, different irrigant agitation techniques and inter-appointment medications.

All these procedures are time-consuming, associated with potential disadvantages and need to be undertaken only if necessary; for example, enlargement of the apical preparation may reduce the effectiveness of the root canal obturation and weaken the root, while interappointment medication is associated with the risk of bacterial leakage of the temporary restoration [19] and increased risk of tooth fracture [18].

A portable, simple to use device, based on the bacterial detection technique described is now being developed.

Authors should discuss the results and how they can be interpreted in perspective of previous studies and of the working hypotheses. The findings and their implications should be discussed in the broadest context possible. Future research directions may also be highlighted.

## 5. Conclusions

The probability of 12-month success was 4.5 times higher when the fluorescence reading was lower than 67 AU, with success rates of 90% and 20%, respectively, for teeth with readings below and above this threshold. 

The fluorescence reading technique provides a surrogate endpoint for RCT and may also allow a reduction in the time required to design new endodontic instruments, devices irrigants and medications, allowing a timely assessment of their effectiveness in disinfecting the RCS, without engaging in expensive and time-consuming clinical trials in the early stages of their development.

## Figures and Tables

**Table 1:** The outcome categories for root canal treatment [12].

Score	Description	Outcome
1	New periapical radiolucency	Unfavourable outcome
2	Enlarged periapical radiolucency	Unfavourable outcome
3	Unchanged periapical radiolucency	Unfavourable outcome
4	Reduced periapical radiolucency	Favourable outcome
5	Resolved periapical radiolucency	Favourable outcome
6	Unchanged healthy periapical status (no radiolucency before and after treatment)	Favourable outcome

**Table 2:** Relationship between the outcome of root canal treatments according to preoperative variables as determined by PR and CBCT.

	Periapical Radiograph		CBCT	
Variable	Number of Teeth	Favourable Outcome	Number of Teeth	Favourable Outcome
Preoperative apical periodontitis				
Absent	8	100% (8)	7	100% (7)
Present	57	87.7% (50)	58	82.8% (48)
Tooth type				
Anterior	13	84.6% (11)	11	84.6% (11)
Premolar	8	87.5% (7)	8	87.5% (7)
Molar	44	90.9% (40)	44	84.2% (37)

**Table 3:** Effect of apical extension of root filling assessed on CBCT images on root canal treatment outcome.

Tooth Type	Inadequate (Short and Long) Favourable Outcome (%)	Adequate Favourable Outcome (%)
Anteriors	100% (2/2)	81.8% (9/11)
Premolars	100% (1/1)	85.7% (6/7)
Molars	42.9% (3/7)	91.9% (34/37)
Total	60% (6/10)	89.1% (49/55)
